# Supplementary material for: Monolithic scalable compliant mechanisms
Source: PLoS One. 2026 Jan 21;21(1):e0340272. doi: 10.1371/journal.pone.0340272 (PMC12822923; doi:10.1371/journal.pone.0340272)
Supplement: S1 Appendix — Information regarding the force/displacement and strain data collection [66], the supplemental movie documentaries, and the derivation of equations is described in this appendix. (DOCX) [file pone.0340272.s001.docx]

Supplementary Text

Data Files

Data S1. Force_Displacement_Data_A1_A2_A5.xlsx

The force/displacement data collected from the A.1, A.2, and A.5 parallel guided mechanisms shown in **Fig. 4A** using the method illustrated in **Fig. 6**.

Data S2. Force_Displacement_Data_B1_B2_B4.xlsx

The force/displacement data collected from the B.1, B.2, and B.4 parallel guided mechanisms shown in **Fig. 4B** using the method illustrated in **Fig. 6**.

Data S3. Strain_Data_A1_A2_A5.xlsx

The strain data collected from the A.1, A.2, and A.5 parallel guided mechanisms shown in **Fig. 4A** using the strain gauge setup shown in **Fig. 7** and the actuation method shown in **Fig. 6**.

The **Data S1**, **S2**, and **S3** files are publicly available in the BYU Scholar’s Archive Repository under the title "Compliant Parallel-Guided Mechanisms Scaling Data (2025)" [41].

Explanatory Text Regarding Force/Displacement and Strain Data

There is force displacement data for each specimen (A.1, A.2, A.5, B.1, B.2, B.4) in the **Data S1** or **Data S2** files. Three repeated tests of each specimen were performed, and these tests are grouped and labeled as X.1-1, X.2-2, and so on. The default units of measurement for the load cell are inches and pound-force. The default units of measurement for the strain gauge data acquisition system are seconds and strain (unitless). The force/displacement data for the Series A parallel guided mechanisms was captured simultaneously with the strain data (**Data S3**) via a separate data acquisition system. This data exhibits a period of time before and after each test where force and strain values are constant. After the data collection, values for strain were mapped to corresponding displacement values based on a clear moment of impact when the probe touches the mechanism and force and strain values begin to increase past ordinary noise.

Supplemental Movie Documentaries

Three movies were produced that highlight the content shared in this paper but are different than traditional videos supporting technical publications in that they are targeted to a broad international audience. They are produced by either Mark Rober's *CrunchLabs* or Brigham Young University's University Communications. Each movie can be viewed on the public video sharing platform *YouTube* and will be described in chronological order with respect to the date of public release. The first movie, “The World's Smallest Nerf Gun Shoots an Ant,” at <https://www.youtube.com/watch?v=9c2NqlUWZfo&t=475s> was produced by Mark Rober in collaboration with researchers at Brigham Young University. It describes the creation of the projectile launching device and the unique challenges of fabricating it on various scales. The research is aimed at a general audience with no technical background. The video has been viewed more than 68 million times and is translated into 10 different languages. Although unusual for a research paper, it also reaches a much larger audience. The second movie, "Mark Rober's tiny Nerf blaster created by BYU engineers" at <https://www.youtube.com/watch?v=dP81TOSrJCc>, was produced by BYU University Communications. It provides a behind-the-scenes look into the research project with an emphasis on the student/professor experience and faith-based learning. The third movie at [https://www.youtube.com/watch?v=Twn_4AW0M6U](https://www.youtube.com/watch?v=Twn_4AW0M6U%20) was produced by Mark Rober and has a portion that highlights an example in the paper (5:15-6:00). This movie highlights the chair made from LET joints and demonstrates how it is created and assembled from a flat sheet of material.

Derivation of Equations

The method for obtaining $F_{max}$ :

The equation for the yield stress of a fixed-guided beam is given on page 415 of [1].

$$S_{y}=\frac{F_{max}Lh}{4I}$$

Rearranging this equation for $F_{max}$ gives

$$F_{max}=\frac{S_{y}bh^{2}}{3L}$$

For a parallel-guided mechanism consisting of two fixed-guided beams,

$$F_{max}=\frac{{2S}_{y}bh^{2}}{3L}$$

The method for obtaining $\delta_{max}$ :

The equation for the maximum deflection for a flexible cantilever beam is given in equation 2.17 on page 29 of [1].

$$\delta_{max}=\frac{2S_{y}L^{2}}{3Eh}$$

A parallel-guided mechanism consists of two fixed-guided beams. A fixed-guided beam has different boundary conditions than a cantilevered beam. However, a fixed-guided beam can be modeled by connecting the free ends together of two half-length cantilevered beams. This means that the deflection for a fixed-guided beam can be described as

$$\delta_{max}=2\left( \frac{2S_{y}\left( \frac{L}{2} \right)^{2}}{3Eh} \right)$$

And simplified as,

$$\delta_{max}=\frac{S_{y}L^{2}}{3Eh}$$
