# Supplementary material for: Monolithic scalable compliant mechanisms
Source: PLoS One. 2026 Jan 21;21(1):e0340272. doi: 10.1371/journal.pone.0340272 (PMC12822923; doi:10.1371/journal.pone.0340272)
Supplement: S2 Appendix — A brief description of the carbon infiltrated carbon nanotube manufacturing process is given. (DOCX) [file pone.0340272.s002.docx]

Supplementary Text

Explanatory Text Regarding Manufacturing Procedure for Carbon Nanotubes

The generalized procedure used to create CICNT mechanisms used in this work is summarized as follows:

1. 30 nm of alumina is deposited on a silicon wafer.
2. Photoresist is spun-on, and photolithography is performed to define a desired pattern.
3. 4 nm of iron is deposited onto the alumina and photoresist.
4. The photoresist is dissolved, thereby removing the iron deposited above it.
5. Carbon nanotubes are ‘grown’ via an ethylene gas flow at 750°C.
6. The carbon nanotube ‘forest’ is infiltrated with carbon via an ethylene gas flow at 850--900°C.
7. The CICNT structure is removed from the substrate.
