# Supplementary material for: Monolithic scalable compliant mechanisms
Source: PLoS One. 2026 Jan 21;21(1):e0340272. doi: 10.1371/journal.pone.0340272 (PMC12822923; doi:10.1371/journal.pone.0340272)
Supplement: Attachment — Submitted filename: Resolution to Review of MSCMs_Aug31.pdf [file pone.0340272.s004.pdf]

Resolution to Review of "Monolithic Scalable Compliant Mechanisms"

We thank the reviewers for their thoughtful comments. We have carefully reviewed each comment and the paper has been modified accordingly. The text below lists each reviewer comment followed by our response and a summary of the changes made in response to the comment.

Journal Requirements

Comment:

1. Please ensure that your manuscript meets PLOS ONE's style requirements, including those for file naming. The PLOS ONE style templates can be found at https://journals.plos.org/plosone/s/file?id=wjVg/PLOSOne_formatting_sample_main_body.pdf and https://journals.plos.org/plosone/s/file?id=ba62/PLOSOne_formatting_sample_title_authors_affiliations.pdf.

Author response:

Manuscript checked for alignment with PLOS ONE's style requirements.

Comment:

2. Please note that PLOS ONE has specific guidelines on code sharing for submissions in which author-generated code underpins the findings in the manuscript. In these cases, we expect all author-generated code to be made available without restrictions upon publication of the work. Please review our guidelines at https://journals.plos.org/plosone/s/materials-and-software-sharing#loc-sharing-code and ensure that your code is shared in a way that follows best practice and facilitates reproducibility and reuse.

Author response:

No code was used that underpins the findings in the manuscript.

Comment:

Thank you for stating the following financial disclosure:

[This work was supported through funds awarded to BDJ by Castle Grayskull, LLC, owned by Mark Rober. Mark Rober did not participate in data collection and analysis. He did participate in study design, the decision to publish, and preparation of the manuscript.].

We note that one or more of the authors is affiliated with the funding organization, indicating the funder may have had some role in the design, data collection, analysis or preparation of your manuscript for publication; in other words, the funder played an indirect role through the participation of the co-authors. If the funding organization did not play a role in the study design, data collection and analysis, decision to publish, or preparation of the manuscript and only provided financial support in the form of authors' salaries and/or research materials, please do the following:

1. Review your statements relating to the author contributions, and ensure you have specifically and accurately indicated the role(s) that these authors had in your study. These amendments should be made in the online form.

2. Confirm in your cover letter that you agree with the following statement, and we will change the online submission form on your behalf:

"The funder provided support in the form of salaries for authors [insert relevant initials], but did not have any additional role in the study design, data collection and analysis, decision to publish,

or preparation of the manuscript. The specific roles of these authors are articulated in the 'author contributions' section.

Author response:

Mark Rober did play a direct role in study design and decision to publish, so the financial disclosure and other statements should not be altered.

Comment:

4. We note that you have a patent relating to material pertinent to this article. Please provide an amended statement of Competing Interests to declare this patent (with details including name and number), along with any other relevant declarations relating to employment, consultancy, patents, products in development or modified products etc. Please confirm that this does not alter your adherence to all PLOS ONE policies on sharing data and materials, as detailed online in our guide for authors http://journals.plos.org/plosone/s/competing-interests by including the following statement: ""This does not alter our adherence to PLOS ONE policies on sharing data and materials." If there are restrictions on sharing of data and/or materials, please state these. Please note that we cannot proceed with consideration of your article until this information has been declared.

This information should be included in your cover letter; we will change the online submission form on your behalf.

Author response:

The competing interest statement should be updated to include the following language, which has also been added to the updated cover letter: "One of the mechanisms presented in this paper as a case study was previously described in U.S. Patent Publication No. US 2025/0102033 A1, entitled "SCALEABLE FLAT IN-PLANE-MOTION MECHANICAL SPRING." This does not alter our adherence to PLOS ONE policies on sharing data and materials."

Comment:

Thank you for stating the following in the Acknowledgments Section of your manuscript:

[The contributions of Terri Bateman, Kevin Cole, David Morgan, Denise Halverson, Carolina Wright, Grant Ogilvie, Bridgette Kemper, Aliya Bascom, James Wade, Hunter Pruett, Natalie Jaques, Luke Gardner, Ivyann Running, Michael Linder, Brooklyn Peters, Jared Erickson, Austin Martel, Lydia Beazer, Trevor Carter, Audrey Christiansen, Davis Wing, Brooklyn Clark, Andrew Geyser, Corinne Jackson, Samuel McKinnon, Lais Oliveira, Felipe Rivera, Spencer Shirley, Jake Sutton, Nathan Coleman, Philip Klocke, Clark Roubicek, Mitchel Skinner, Katie Varela, and Kyle Dahl, for their work in analysis, prototype fabrication, and testing, are greatly appreciated. We acknowledge Robert Davis and Richard Vanfleet for their work in developing carbon nanotube fabrication technology that was used in this project. Other contributions to manufacturing include Boston Micro Fabrication, with micro-3D printing; FineArc Welding LLC with waterjet cutting; and the Center for Medical Innovation at the University of Utah with Fused Deposition Modeling of nylon parts. We thank BYU Photo for photography services. Funds for this work were provided by Castle Grayskull, LLC.]

We note that you have provided funding information that is **not currently declared in your Funding Statement**. However, funding information should not appear in the Acknowledgments section or other areas of your manuscript. We will only publish funding information present in the Funding Statement section of the online submission form.

Please remove any funding-related text from the manuscript and let us know how you would like to update your Funding Statement. Currently, your Funding Statement reads as follows:

[This work was supported through funds awarded to BDJ by Castle Grayskull, LLC, owned by Mark Rober. Mark Rober did not participate in data collection and analysis. He did participate in study design, the decision to publish, and preparation of the manuscript.]

Please include your amended statements within your cover letter; we will change the online submission form on your behalf.

Author response:

Funding information has been removed from the Acknowledgments section. There was no funding information in the Acknowledgments that was not already included in the funding statement, so the funding statement should not be altered.

Comment:

6. We note that Figure 1 and 9 includes an image of participant in the study.

As per the PLOS ONE policy (http://journals.plos.org/plosone/s/submission-guidelines#loc-human-subjects-research) on papers that include identifying, or potentially identifying, information, the individual(s) or parent(s)/guardian(s) must be informed of the terms of the PLOS open-access (CC-BY) license and provide specific permission for publication of these details under the terms of this license. Please download the Consent Form for Publication in a PLOS Journal (http://journals.plos.org/plosone/s/file?id=8ce6/plos-consent-form-english.pdf). The signed consent form should not be submitted with the manuscript, but should be securely filed in the individual's case notes. Please amend the methods section and ethics statement of the manuscript to explicitly state that the patient/participant has provided consent for publication: “The individual in this manuscript has given written informed consent (as outlined in PLOS consent form) to publish these case details”.

If you are unable to obtain consent from the subject of the photograph, you will need to remove the figure and any other textual identifying information or case descriptions for this individual.

Author response:

Consent forms have been obtained for each individual present in photographs in the paper and will be retained by the authors for their own files. Added statements to the captions of Figures 1 and 9 stating that “The individual in this figure has given written informed consent (as outlined in PLOS consent form) to have their image appear in this paper.” The ethics statement was also amended to say: “N/A: No human subject, animal, or field research was performed. However, the individuals who appear in Figures 1 and 9 have given written informed consent (as outlined in PLOS consent form) to have their image appear in this paper.”

Comment:

7. Thank you for stating the following in the Competing Interests section:

[I have read the journal's policy and the authors of this manuscript have the following competing interests: This work was supported through funds awarded to BYU by Castle Grayskull, LLC, owned by Mark Rober. A patent application is pending on the planar spring that is integral to the blaster case study. A patent has been issued on the LET joints that are part of the chair case study.].

We note that one or more of the authors are employed by a commercial company: name of commercial company.

1. Please provide an amended Funding Statement declaring this commercial affiliation, as well as a statement regarding the Role of Funders in your study. If the funding organization did not play a role in the study design, data collection and analysis, decision to publish, or preparation of the manuscript and only provided financial support in the form of authors' salaries and/or research materials, please review your statements relating to the author contributions,

and ensure you have specifically and accurately indicated the role(s) that these authors had in your study. You can update author roles in the Author Contributions section of the online submission form.

Please also include the following statement within your amended Funding Statement.

"The funder provided support in the form of salaries for authors [insert relevant initials], but did not have any additional role in the study design, data collection and analysis, decision to publish, or preparation of the manuscript. The specific roles of these authors are articulated in the 'author contributions' section."

If your commercial affiliation did play a role in your study, please state and explain this role within your updated Funding Statement.

2. Please also provide an updated Competing Interests Statement declaring this commercial affiliation along with any other relevant declarations relating to employment, consultancy, patents, products in development, or marketed products, etc.

Within your Competing Interests Statement, please confirm that this commercial affiliation does not alter your adherence to all PLOS ONE policies on sharing data and materials by including the following statement: ""This does not alter our adherence to PLOS ONE policies on sharing data and materials." (as detailed online in our guide for authors http://journals.plos.org/plosone/s/competing-interests) . If this adherence statement is not accurate and there are restrictions on sharing of data and/or materials, please state these. Please note that we cannot proceed with consideration of your article until this information has been declared.

Please include both an updated Funding Statement and Competing Interests Statement in your cover letter. We will change the online submission form on your behalf.

Author response:

The updated Funding and Competing Interest Statements were added to the cover letter.

The updated Funding Statement is as follows: "This work was supported through funds awarded to BDJ by Castle Grayskull, LLC, founded by Mark Rober. The funder did not participate in data collection and analysis, but did provide support in the form of salaries for authors JH, BP, and JS and was involved in the study design, decision to publish, and preparation of the manuscript. The specific roles of these authors are articulated in the 'author contributions' section."

The full Competing Interests Statement should now read as follows: "I have read the journal's policy and the authors of this manuscript have the following competing interests: This work was supported through funds awarded to BYU by Castle Grayskull, LLC, owned by Mark Rober. These funds supported authors JH, BP, and JS in the form of salaries. Mark Rober is also one of the authors of this paper and was involved in the study design, decision to publish, and preparation of the manuscript. A patent application is pending on the planar spring that is integral to the blaster case study. A patent has been issued on the LET joints that are part of the chair case study. Another of the mechanisms presented in this paper as a case study was previously described in U.S. Patent Publication No. US 2025/0102033 A1, entitled "SCALEABLE FLAT IN-PLANE-MOTION MECHANICAL SPRING." This does not alter our adherence to PLOS ONE policies on sharing data and materials."

Comment:

8. If the reviewer comments include a recommendation to cite specific previously published works, please review and evaluate these publications to determine whether they are relevant and

should be cited. There is no requirement to cite these works unless the editor has indicated otherwise.

Author response:

A citation was added to the reviewer's suggested work.

Reviewer #1

Comment:

The manuscript presents an investigation into the scalability of displacement-driven compliant mechanisms, addressing an important aspect of compliant mechanism design that has significant implications for multi-scale applications. The authors demonstrate, through experimental validation and detailed case studies, that the strain remains constant across geometric scales while force scales linearly with width, confirming the theoretical foundations proposed.
The discussion appropriately highlights the counterintuitive nature of displacement-driven compliant mechanisms compared to conventional force-driven systems, and, in general, it insightfully points to potential future applications.
Overall, the topic fits the interest of the journal.
Language and writing, under the syntactic and grammar aspects, results in being sufficiently fluent and clean.

Author response:

Thank you for the review.

Comment:

1) The manuscript could benefit from a more detailed exploration of potential manufacturing challenges and material limitations that might arise at extreme scales, as well as a clearer explanation of how these scaling principles might integrate with existing design optimization frameworks.

Author response:

These discussions about manufacturing and the design process would enrich the paper and provide greater benefits to the reader trying to apply scaling principles to their own work.

Author action:

To address the exploration of potential manufacturing challenges and material limitations, a paragraph was added to the "Case Study: Scaling Beams in Bending" section, as follows: "When manufacturing at extreme scales, such as the C = 0.01 and C = 7.25 scales of this projectile launcher, some difficulties arise. For example, at the microscale, the smallest feature of the projectile launcher is less than 100 microns. Manufacturing long slender beams for bending with this width while avoiding failure, even with CICNTs, can be difficult and requires extreme precision and carefully selected parameters in manufacturing (see updated paper for citations). This is also linked to material limitations: research in the area of improving mechanical properties of CICNTs often focuses on increasing strength and modulus (citations), while compliant mechanisms applications benefit instead from a lower modulus (citations). Additionally, manufacture at the micro scale can be more time and resource intensive than at the macro scale. On the mega scale, in extreme cases, it can become difficult to obtain sheet material or manufacturing facilities large enough to create the mechanism in a single piece, without requiring assembly. This can also become more time and cost intensive."

A discussion of the integration of scaling principles into existing design optimization frameworks was also added to the Discussion section: "Existing compliant mechanisms design frameworks typically include a definition of the desired behavior, concept selection, virtual prototyping,

followed by physical prototyping (citations). Without knowledge of the scaling principles presented here, this design process would essentially need to be performed once for each scale of the mechanism. By implementing scaling principles into this existing framework during the first step of the design process, designers can instead perform the design process a single time for multiple scales and would need to consider tuning for the manufacturing process and material properties of the resultant design to achieve performance at differing scales. This has the added benefit of simplifying prototyping for extreme scales by allowing it to be done primarily at a macro scale, where lessons learned from can then be applied when manufacturing the final products in the micro or mega scales."

Comment:

2) The explanation of the theoretical background, while generally sound, lacks sufficient depth and rigor in some parts; for example, the assumptions behind the scaling laws are not always clearly stated or justified, which may leave readers questioning the generality of the results.

Author response:

We appreciate the reviewer's note. The scaling laws are applicable to displacement-driven compliant mechanisms in general. We have modified the introduction to clarify this and have added text to the "Making Stress Independent of Scale" section.

Author action:

To clarify the generality of the results, the introduction was modified and the following text was added to the "Making Stress Independent of Scale" section: "The scaling factors presented in Table X were directly derived for the parallel-guiding mechanism, which has fixed-guided boundary conditions and experiences an applied deflection at the end of the beams. While the Euler-Bernoulli beam theory equations are slightly altered based on the boundary and loading conditions, when dividing the model representing the scaled geometry by the model representing the unscaled geometry, the changes cancel, leaving the same resultant scale factors. These scaling factors are therefore applicable to displacement-driven compliant mechanisms in general." Additional references were also added to Ref. [1], which provides an analytical basis for eqs. (1) and (2) (now eqs. 3 and 6), and derivation and explanation for these equations was added from Appendix S1 to the main text.

Comment:

3) The discussion section touches on promising applications but tends to be somewhat speculative without clear pathways for practical implementation or addressing potential limitations in manufacturing and material behavior at extreme scales.

4) Furthermore, the paper would benefit from a more critical treatment of challenges related to scaling, such as non-ideal material properties, fabrication tolerances, and environmental factors.

5) The paper also overlooks the possible impact of stress concentrations responses when scaling compliant mechanisms, which could lead to unexpected failure modes (or early failure) during the application of cycles loads.

6) How does fatigue behavior modify when dimensions change? Please, add a discussion on this point in the manuscript.

Author response:

Readers would benefit from having a more thorough understanding of how to practically apply these scaling principles to their research and designs.

Author action:

Information on the a) manufacturing and material behavior at extreme scales and b) integration of scaling principles into existing design frameworks was added to the first case study and the discussion, respectively, as described above.

The following discussion was also added to the Discussion section to address these points:

"When scaling displacement-driven compliant mechanisms, it is also important to consider challenges for manufacturing at micro or mega scales. Manufacturing at the micro-scale is possible through limited methods and is typically slower, more expensive, and requires more complex systems than at the macro-scale (citations), particularly compared to macro-scale rapid prototyping methods. For complex systems that are expected to achieve motion through compliant members, such as the toy projectile launcher discussed in the case study above, the micro-scale manufacturing options become even more limited (citations). Fabrication tolerances also scale approximately with the mechanism, so what might start as a tolerance of 1 mm at the C = 1 scale might become a tolerance of a 10 microns at the C = 0.01 scale, which again increases manufacturing difficulty.

It is also important to consider non-ideal material properties and the potential for stress concentrations when working with extreme scales. A tiny material nonideality or stress concentration that might in a certain material or scale not affect performance could in another material or scale decrease performance or even lead to catastrophic failure. Additionally, for mechanisms undergoing repeated cyclic motion, which compliant mechanisms often require, a consideration of fatigue behavior is also important. As with stress concentrations, we see similarly magnified effects in fatigue life: what could have a minimal effect in one material or scale, might significantly reduce fatigue life in another material or scale."

Comment:

7) Authors may consider discussing the following reference: in this recent paper on cross-axis flexure hinges (a) https://doi.org/10.1016/j.mechmachtheory.2024.105894, the authors demonstrate that second-order kinematic invariants (specifically the polodes and the inflection circle) scale linearly with the length of the flexure hinge. This finding offers an interesting perspective that could enrich your discussion by highlighting that certain elasto-kinematic properties of compliant mechanisms also exhibit scaling relationships useful for the design.

Author response:

This is an interesting and relevant paper.

Author action:

A citation to this paper was added.

Reviewer #2

Comment:

This work shows that for displacement-driven compliant mechanisms, mechanical stress is uniquely invariant with scale. This effect is described theoretically, verified through computer models and physical testing, and is demonstrated in three examples: a parallel-guiding mechanism, a projectile launcher, and a chair.

The work is useful and emphasized scalability challenges and how to overcome them.

Author response:

Thank you for your review.

Comment:

- the motivation can be improved on why scalability matters and how to approach it. In the case of compliant mechanisms how scalability can be beneficial for monolithic design

Author response:

The motivation of scalability as well as some suggested considerations for designers will help readers place the research into context.

Author action:

The following was added to the Introduction section: "Through understanding principles of scalability, designers can design and prototype initially for the macroscale, where prototyping is simpler and may be performed more quickly (e.g. through use of 3D printers or traditional prototyping methods), before easily scaling up or down their design for the final iterations. Since manufacturing at extreme scales is often more complex and expensive (citations), understanding scaling principles has the potential to improve the design process for the applications of interest as listed above."

Comment:

- discuss the actuation of complaint mechanisms as well as the power needed in the context of scalability

Author response:

Power in the context of scalability is an interesting consideration, especially when non-manual methods are required to actuate the mechanism.

Author action:

The following was added to the "Making Stress Independent of Scale" section: "Compliant mechanisms may be actuated through a variety of methods (citations), including cable-driven, shape-memory alloy, piezoelectric, magnetic, fluidic, pneumatic, or resorbable materials (citations). For the mechanisms developed here, we generally used manual methods, e.g. actuation through manual application of deflection. Regardless of the actuation method selected, since smaller-scale mechanisms experience decreased stiffness, potential energy, and total deflection (as shown in Table X), they also require less power to experience their full actuation."

Comment:

- the conclusions need improvement: clearly state the contributions of the work as well as how to interpret the main findings for design

Author response:

Readers would benefit from having a more thorough understanding of how to practically apply these scaling principles to their research and designs.

Author action:

Significant discussion was added to the Discussion section on how designers might practically apply this to their work, including a discussion of when to implement scaling considerations into the compliant mechanisms design process and challenges when manufacturing at extreme scales. The conclusion was also improved with the following addition:

"Scaling a physical device's geometry results in mechanical properties changing in various ways (e.g. the cubed-squared law states that for a scaling factor C, mass scales with C^3 and surface area with C^2. These scaling effects can result in a device's inconsistent and unplanned mechanical behavior when varying its fabricated size, thereby necessitating unique designs at different scales. We show that for displacement-driven compliant mechanisms, mechanical stress is uniquely invariant with scale. This effect is described theoretically, verified through computer

models and physical testing, and is demonstrated in three examples: a parallel-guiding mechanism, a projectile launcher, and a deployable chair. This enhanced understanding of stress invariance provides innovative insight into the way devices can be designed for systems that operate across different scales."
