# Supplementary material for: Monolithic scalable compliant mechanisms
Source: PLoS One. 2026 Jan 21;21(1):e0340272. doi: 10.1371/journal.pone.0340272 (PMC12822923; doi:10.1371/journal.pone.0340272)
Supplement: Attachment — Submitted filename: 2nd Resolution to Review of MSCMs_Nov4 .docx [file pone.0340272.s005.docx]

Resolution to Review of “Monolithic Scalable Compliant Mechanisms”

We thank the reviewers for their further thoughtful comments. We have carefully reviewed each comment and the paper has been modified accordingly. The text below lists each reviewer comment followed by our response and a summary of the changes made in response to the comment.

**Reviewer #3**

Comment:

All my indications and/or suggestions have been correctly implemented. The paper is well written and well structured, and the results have been described in a thorough and comprehensive manner. I believe that the paper is ready for publication.

Author response:

Thank you for the review!

**Reviewer #4**

Comment:

This manuscript explores the scalability of displacement-driven compliant mechanisms, demonstrating that mechanical stress remains invariant under geometric scaling. The work combines analytical derivation, finite element analysis, and physical testing across three illustrative case studies: a parallel-guiding mechanism, a projectile launcher, and a deployable chair. The authors argue that this property simplifies design transferability across scales and opens new opportunities in areas such as MEMS, medical devices, and deployable structures.
The revised manuscript addresses most of the issues raised in earlier review rounds, improving clarity, rigor, and relevance. Overall, it represents a valuable and well-supported contribution to the field of compliant mechanism design. The following comments need to be addressed.

Author response:

Thank you for the review!

Comment:

1. Add scale bar in Fig. 1, Fig. 3. Also Fig. 1 a, a and c and be laid in single column.

3. Figure 3 and 4 can be merged to single figure as they are similar and this will make the manuscript more readable.

Author response:

- Scale bars have been added to Figure 1 a, b, c. The subfigures are now laid out in a single column.

- Figure 3 has been merged with Figure 4 and scale bars have been added.

- Figure 5 has been renamed Figure 4. Similarly, Figure 8 was renamed as Figure 6 and Figure 9 as Figure 7.

- Figure references and captions were updated.

Comment:

2. Figure 2 sub figures should be labeled as a, b, c and d to improve readability

Author response:

- Figure 2 subfigures are now labeled a, b, c, and d to improve readability.

Comment:

4. Did author check the projectile launch for all scales? What the distance that the launcher can travel as illustrated in Figure 9. Is MEMS launcher functional as beans are deformed. Can it store the force, it should be supported with video or tracking the displacement.

Author response:

An approximate projectile launch distance for each scale has been added to the end of the “Case study: Scaling beams in bending” section:

“The *C* = 1 scale additively manufactured launcher shown in Fig 7C is able to launch the dart approximately 4 m from a standing position. The CICNT 1/10th scale as shown in Fig 7 is able to launch the dart approximately 10 cm from a position flat on the table. The CICNT 1/100th scale is able to launch the dart approximately 3 cm from flat on the table. An example of each of these sizes launching, including visuals of the torsional beams in deformation, is available in ([Rober, 2023](https://www.youtube.com/watch?v=9c2NqlUWZfo&t=456s)).”

Comment:

5. Figure 6 and 7 should be merged as they don’t add more information. How were the sensors on Figure 7 calibrated?

Author response:

- Figures 6 and 7 have been merged into one to create Figure 5.

A description of the sensor calibration was added to the “Experimental validation of scaled parallel-guiding mechanism” section:

“A physical calibration method was used to ensure accurate load cell measurements. The load cell was first connected to the Data Acquisition system (DAQ) and zeroed. A dead weight of known magnitude was placed on the load cell to confirm the measurement was as expected. The tensile tester was rotated such that the direction of travel was perpendicular to gravity. A lightweight probe was attached to the load cell and applied a small moment to it, but the structural stiffness of the load cell rendered the effect too slight to consider. The load cell was kept at the same calibration state throughout trials; there were no software calibrations in between tests on the parallel-guided mechanism specimens.”

Comment:

6. A supporting video on formation of chair should be included in the supporting information along with projectile launch.

Author response:

A supporting video on the chair has been added to the supplemental material (S3 Appendix).

Comment:

7. Add scale bar for Figure 10. What weight of the person that the chair can bear without deformation or break.

Author response:

- Figure 10 was renamed as Figure 8 and a scale bar was added.

A description of the chair’s load-bearing capabilities were added to the “Case study: Scaling beams in torsion” section:

“Parkinson et al. performed modeling on the birch plywood chair which demonstrated that it could withstand 4,000 N before failure. The acrylic version is expected to be able to withstand a lower force but is verified for at least 1,000 N through usability testing.”

Comment:

8. The following statement should be supported by experimental results: ‘A Keyence VHX microscope was used in conjunction with a micromanipulator probe to view and actuate the CICNT devices’

Author response:

This statement was expanded to include a reference to the Rober, 2023 video. This video shows the actuation of the 1/100^th^ scale CICNT device through the methods described.

“A Keyence VHX microscope was used in conjunction with a micromanipulator probe to view and actuate the CICNT devices, as shown in video for the 1/100th scale in (Rober, 2023).”

Comment:

9. Recent work on compliant mechanisms with multi degrees of freedom (DOF) should be included in discussion section. Some examples includes.
i. Yang, H., Patel, D.K., Johnson, T. et al. A compliant metastructure design with reconfigurability up to six degrees of freedom. Nat Commun 16, 719 (2025). <https://doi.org/10.1038/s41467-024-55591-2>
ii. Humphrey Yang, et al. 2022. ReCompFig: Designing Dynamically Reconfigurable Kinematic Devices Using Compliant Mechanisms and Tensioning Cables. In Proceedings of the 2022 CHI Conference on Human Factors in Computing Systems (CHI '22). Association for Computing Machinery, New York, NY, USA, Article 170, 1–14. <https://doi.org/10.1145/3491102.3502065>

Author response:

An addition was made to the “Making stress independent of scale” section making reference to these works:

“The degrees of freedom of a compliant mechanism would also remain constant as it is scaled (Yang 2022, Yang 2025, Yu 2025).”
